# The Congresses

**Published:** 1897-09-11

**Authors:** 


Vol. XXII.—No. 572.] [Sept. 11, 1897.

### The Congresses.

Another season of congresses is drawing to a close, and it does not seem unreasonable to ask, What is the use of all this travel, and wherein does man profit by all this talk? We fear that there are many, now counting up the cost, who will echo, somewhat sadly, What indeed? It has, in fact, to be confessed that the social gratification of being a unit in a vast congress is not extreme, while the scientific advantage is infinitesimal, except to the few within the circle. When we hear that 7,000 members attended the Moscow Congress we can readily understand not only the difficulties and discomforts to which many of them were exposed, but the absolute impossibility of rendering the scientific advantages of such a meeting accessible to even a tithe of its nominal members. But not only have congresses of late been rendered more or less unmanageable by mere numbers, but it is to be feared that the recognition of the vast diversity of mind existing in so great a multitude has had a deteriorating influence on the addresses prepared for their delectation. The very interesting meeting of the British Association was largely saved from this defect, for "science" is so wide a subject that its devotees are naturally divided into sections, each of which is largely independent of the rest. Thus it happened that in almost every case the distinguished men who gave the addresses spoke out of their own knowledge, and gave the results of their own experience.

At Montreal things were different. Medicine is maintained by its professors to be one and indivisible, and although at the meeting of the British Medical Association the subjects were spread over eleven sections, the readers of the addresses, in endeavouring to be interesting to all men, lost to some extent that intimate touch with their own experience and their own life work which is the secret of success. This is no new thing. It has become the fashion for presidents of sections to dive down into the past, and, from such uncertain data as they can there discover, to trace the progress of their science down to the actualities of the present day. We cannot say that the result is entirely satisfactory. Men are appointed to prominent positions in scientific congresses on account of their eminence in scientific work, without the smallest reference to their capacity as historians, and we cannot but feel that, in addressing an assembly of medical men drawn from all parts of the world, a physician of wide experience wastes an opportunity which can only rarely fall to any man's lot, if, instead of speaking out of the stores of knowledge gained in his own hospital, and by his own work, he delivers an address on some historical topic obviously got up for the occasion. What happened at Montreal was that the representative of medicine, that most progressive science—and not the medicine of a slow conservative country, but of perhaps the most progressive nation on the globe—turned aside from problems of the day as if they were not, put behind him the experience of his life and of his hospital as if such were of no importance, and, plunging into the "dim dawn of history," led up, with much art and literary industry, no doubt, to a history of British medicine in Greater Britain. The whole address was a graceful testimony to the unity of the English-speaking race. It was a kindly thought which led the Professor of Medicine in one of the greatest medical schools of the United States thus to hold out the warm hand of brotherhood, adding the welcome of his great country to that of Canada. Moreover, the address was admirable in every way and most acceptable to Britons; but to many who were present it must have been a disappointment to find that instead of Osler the physician, whom they had come to hear, Osler the historian occupied the tribune. Nor did any different fate befall the surgeons who went to listen to words of wisdom from the mouth of Mitchell Banks of Liverpool. The name of Mitchell Banks is associated in the minds of most men with bold and successful surgery, and with important and life-saving advances in operative methods; but, when set to find a subject for an address, he also must needs dive into history and treat of the Surgeon of Old in War. Not that we grumble, for his address, like everything he writes, was of a most interesting character throughout. We mention it merely as another illustration of the tendency of large congresses to tempt men into the vague and uncertain paths of history instead of giving to their colleagues the valuable results of their own personal experience.

Enough, however, of criticism. Science is not everything, and we must remember that to the mass these congresses are the one great holiday of the year. The picnics, the excursions, the garden parties and social functions of all kinds, the travel in strange countries, the unaccustomed routine, the wiping out of many insular prejudices and conceits, all tend to make the member of a congress a better, a more tolerant, and a broader-minded man than he was when he packed up his traps and took his ticket.

So far as the majority are concerned this is the chief benefit of a congress, and it is surely no small gain to humanity that those who from their position must always be teachers, and, in a way, leaders of men, should be saved from falling into ruts.

